# Body‐Integrated Ultrasensitive All‐Textile Pressure Sensors for Skin‐Inspired Artificial Sensory Systems

**DOI:** 10.1002/smsc.202400026

**Published:** 2024-06-30

**Authors:** Bingjun Wang, Yuanhong Shi, Haotian Li, Qilin Hua, Keyu Ji, Zilong Dong, Zhaowei Cui, Tianci Huang, Zhongming Chen, Ruilai Wei, Weiguo Hu, Guozhen Shen

**Affiliations:** ^1^ Beijing Institute of Nanoenergy and Nanosystems Chinese Academy of Sciences Beijing 101400 China; ^2^ School of Nanoscience and Technology University of Chinese Academy of Sciences Beijing 100049 China; ^3^ School of Integrated Circuits and Electronics Beijing Institute of Technology Beijing 100081 China; ^4^ Institute of Flexible Electronics Beijing Institute of Technology Beijing 102488 China

**Keywords:** artificial sensory systems, pressure sensors, skin‐inspired, textile, ultrasensitive

## Abstract

Tactile sensing plays a vital role in human somatosensory perception as it provides essential touch information necessary for interacting with the environment and accomplishing daily tasks. The progress in textile electronics has opened up opportunities for developing intelligent wearable devices that enable somatosensory perception and interaction. Herein, a skin‐inspired all‐textile pressure sensor (ATP) is presented that emulates the sensing and interaction functions of human skin, offering wearability, comfort, and breathability. The ATP demonstrates impressive features, including ultrahigh sensitivity (1.46 × 10^6^ kPa^−1^), fast response time (1 ms), excellent stability and durability (over 2000 compression‐release cycles), a low detection limit of 10 Pa, and remarkable breathability (93.2%). The multipixel array of ATPs has been proven to facilitate static and dynamic mapping of spatial pressure, as well as pressure trajectory monitoring functions. Moreover, by integrating ATP with oscillation circuits, external force stimuli can be directly encoded into digital frequency pulses that resemble human physiological signals. The frequency of output pulses increases with the applied pressure. Consequently, an ATP‐based artificial sensory system is constructed for intelligent tactile perception. This work provides a simple and versatile strategy for practical applications of wearable electronics in the fields of robotics, sports science, and human–machine interfaces technologies.

## Graphical abstract

A skin‐inspired all‐textile pressure sensor (ATP) is designed to mimic the sensing and interaction functions of human skin. The ATP operates in a pressure‐triggered frequency encoding mode and delivers an ultrahigh sensitivity of 1.46 × 10^6^ kPa^−1^, offering wearability, comfort, and breathability. The multipixel ATP‐based array enables static and dynamic mapping of spatial pressure while facilitating the monitoring of pressure trajectories.

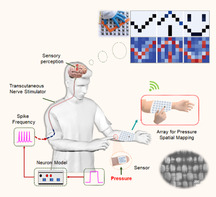

## 1 Introduction

The human somatosensory system plays a crucial role in perceiving external stimuli and converting them into physiological signals that are transmitted to the brain through the nervous system.^[^ ^1^, ^2^, ^3^, ^4^, ^5^ ^]^ This process enables humans to respond differently to various stimuli. Touch, as a vital sense of the somatosensory system, allows us to gather information and learn from our environment through continuous physical interactions.^[^ ^6^ ^]^ Simulating such human interactions not only contributes to the understanding of human behavior, but also drives the development of innovative interactive devices that enhance human experiences in entertainment, education, and healthcare.^[^ ^7^, ^8^ ^]^ In recent years, the progress of wearable technology, coupled with the growing demand for flexible, deformable, and easy‐to‐fabricate electronic devices, has led to significant advancements in textile‐based sensors.^[^ ^9^ ^]^ These sensors offer flexibility, comfort, breathability, durability, and sewability, overcoming the limitations of poor air permeability and comfort associated with sensors based on ultrathin substrates like polyimide, polydimethylsiloxane, and silicone.^[^ ^10^, ^11^, ^12^, ^13^, ^14^ ^]^ Consequently, textile‐based sensors have emerged as ideal choices for creating natural and nonintrusive interactive devices. The development of these sensors has the potential to revolutionize the field of wearable electronics, introducing a new dimension of interactivity between humans and machines. By leveraging the advantages of textile‐based sensors, such as comfort and breathability, we can create innovative devices that seamlessly integrate with our daily lives and enhance our interactions with technology. This opens up exciting possibilities for the future of wearable electronics and its impact on various domains.

High‐performance pressure sensors equipped with tactile sensing capabilities can serve as the fundamental building blocks for implementing applications involving skin‐inspired artificial perception and interaction.^[^ ^15^, ^16^ ^]^ Currently, various types of pressure sensors, including resistive,^[^ ^10^, ^17^, ^18^ ^]^ capacitive,^[^ ^19^, ^20^ ^]^ piezoelectric,^[^ ^21^, ^22^, ^23^ ^]^ and optoelectronic,^[^ ^24^ ^]^ have been extensively studied. Among them, resistive pressure sensors offer distinct advantages in terms of their sensitivity, reliability, and cost‐effectiveness. To enhance device performance, researchers have explored novel materials and structures (e.g., micropillar,^[^ ^25^, ^26^, ^27^ ^]^ micropyramids,^[^ ^26^, ^28^, ^29^ ^]^ or other biomimetic patterns^[^ ^30^, ^31^, ^32^, ^33^ ^]^) to optimize pressure sensors. In addition to pursuing high‐performance improvements, it is crucial to explore practical and scalable applications of textile‐based pressure sensors. For instance, integrating textile tactile sensors into clothing enables equipping humanoid robots with electronic skin for effective human–robot collaboration.^[^ ^16^ ^]^ These sensors can also be fabricated as wearable fabrics worn on different parts of the human body to facilitate health monitoring.^[^ ^15^, ^20^ ^]^ Moreover, the growing prominence of virtual reality (VR) technology underscores the potential of textile sensors in enhancing human experiences.^[^ ^34^ ^]^ To achieve a level of perception and feedback comparable to or surpassing that of human skin, it is essential to establish a direct connection between the sensor and the human nervous system. This involves converting the analog signals generated by the sensor into physiological signals, referred to as action potentials, which can be recognized by human neurons.^[^ ^35^ ^]^ While studies have demonstrated that force stimulation can be encoded into digital frequency signals similar to human pulse waveforms through oscillation circuits,^[^ ^36^, ^37^, ^38^, ^39^ ^]^ further research is required to develop an integrated sensory system that incorporates the sensor's stimulus sensing and neural information encoding functions. Therefore, exploring the application of high‐performance textile‐based pressure sensors in wearable intelligent perception and interaction holds significant practical significance.

In this work, we report a novel wearable all‐textile pressure sensor (ATP) for skin‐inspired intelligent sensory systems. The piezoresistive‐type pressure sensor is fabricated using an electroless plating process of nickel on textiles, which is well established and easy to perform with no need of electrical power, and exhibits super bonding strength with the textile substrate compared to electroplating. The resulting coating offers advantageous characteristics, including uniform and controllable thickness, high hardness, and strong corrosion resistance, making it highly suitable for nickel plating on textile surfaces. ATP demonstrates exceptional performance with ultrahigh sensitivity (1.46 × 10^6^ kPa^−1^), ultrafast response speed (1 ms), superior stability and durability (2000 compression‐release cycles), and low detection limit (10 Pa). The fabric‐based sensors are flexible, comfortable, and breathable, which enables them to perfectly conform to different parts of the human body. An ATP‐based 7 × 7 pressure sensor array is woven to achieve spatial pressure mapping and real‐time pressure trajectory monitoring. Furthermore, by integrating the ATP with an oscillation circuit and a waveform conversion circuit, the pressure stimulus can be directly converted into a digital frequency pulse similar to human physiological signals. Finally, the integration of the ATPs array with the signal conversion circuit successfully demonstrates a smart interactive sleeve that can realize skin‐like sensing and interaction functions. This all‐textile‐based high‐sensitivity pressure sensor may open new avenues for upgrading in fields such as healthcare, human–machine interaction, sports science, robotics, and security monitoring.

## 2 Results and Discussion

**Figure 1:**
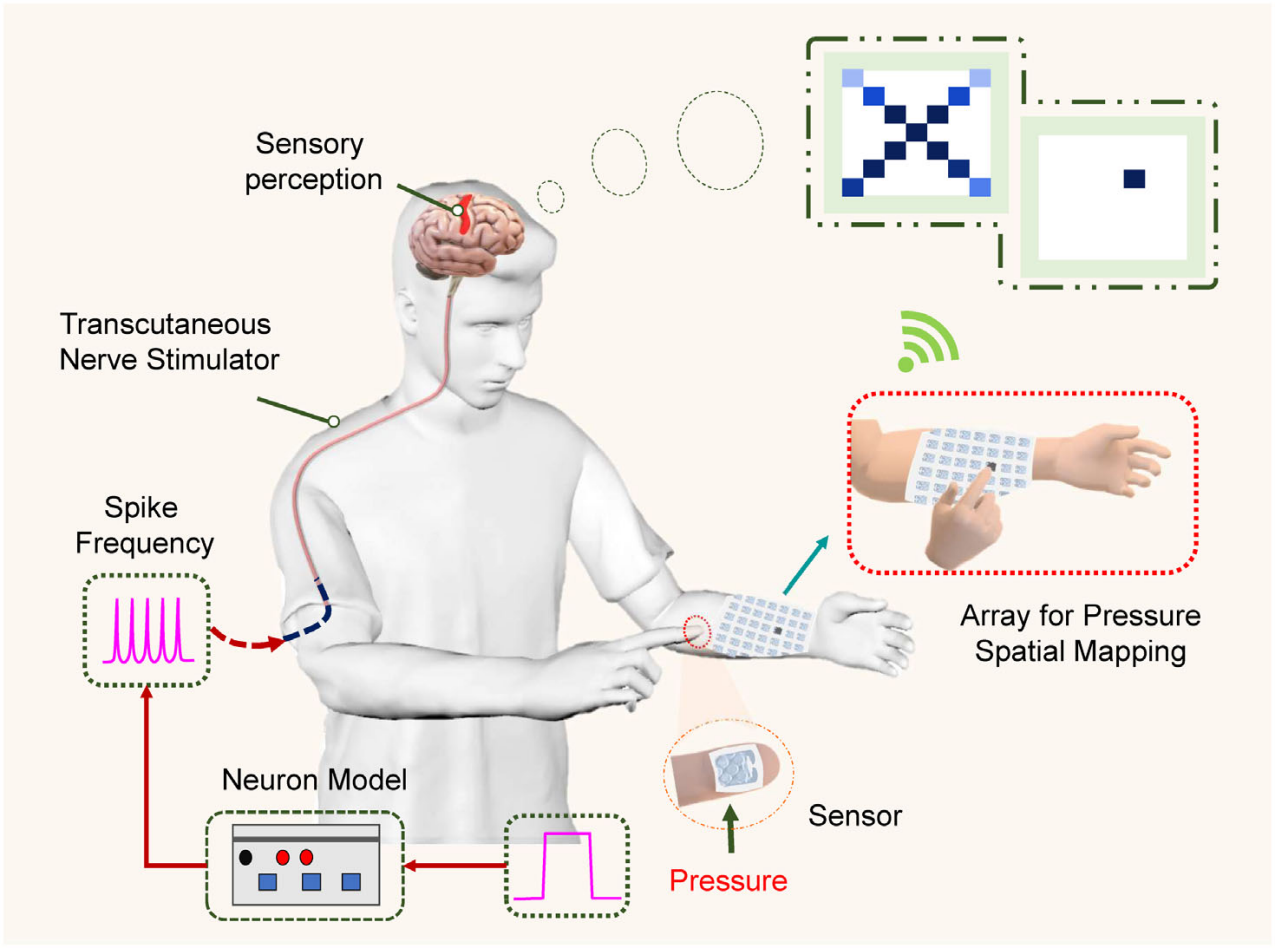
Schematic illustration of skin‐inspired artificial sensory systems for pressure‐sensitive spike encoding and spatial pressure mapping.

**Figure** **1** illustrates a novel concept of an intelligent skin‐like interaction device based on ATP. As a crucial organ of the human body, human skin (such as fingertips) can sense pressure stimuli during touch and these stimuli are transmitted to the nerve cells beneath the skin and converted into corresponding physiological signals (referred to action potentials). Recently, tactile sensor arrays have been successfully developed with the help of advanced micro/nanofabrication technology.^[^ ^40^, ^41^ ^]^ The sensitivity and resolution of some sensors have exceeded that of human fingertip skin.^[^ ^42^, ^43^, ^44^, ^45^ ^]^ However, the large‐scale use of tactile sensor arrays is still a challenging problem. This is because the number of sensor units will increase significantly with the increase of coverage area, and the computing scale of signal processing will also increase significantly. In addition, a large number of distributed wires not only easily produce electromagnetic noise, but the wires can also reduce the robustness and mechanical properties of sensors, such as flexibility and stretchability. Therefore, developing a macroscale sensor with large sensing area is helpful to solve the above limitations of tactile sensor arrays. These signals are then transmitted through the nervous system to the brain, enabling humans to communicate and interact with the external environment. Inspired by the human skin, an all‐textile‐based pressure sensor is introduced to demonstrate the sensation and interaction capabilities. Due to its soft, thin, and breathable advantages, the ATPs can be easily worn on various parts of the human body. Based on the size and location of external stimuli, the ATP‐based sensing system can achieve dynamic spatial pressure mapping and pressure trace monitoring. Furthermore, the encoding of stimuli into physiological signals through integrated signal conversion circuits makes it possible to realize human–computer interaction in the true sense.

**Figure 2:**
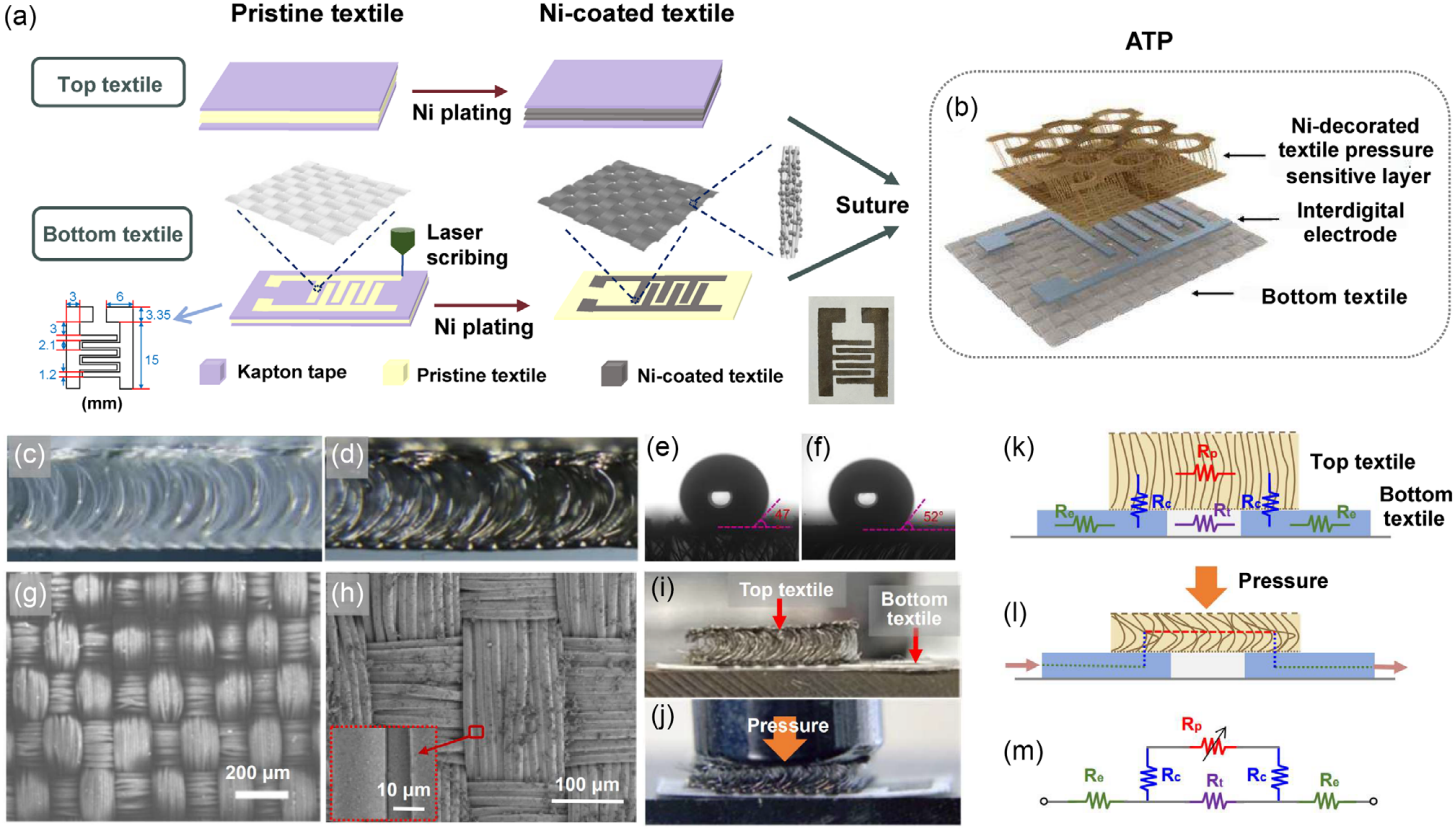
Device characterizations of ATPs. a) Preparation process of the ATP, including the pressure‐sensitive layer of top textile and interdigitated electrodes of bottom textile. b) Schematic illustration of device stack layers, including bottom textile, interdigital electrode, and Ni‐decorated pressure‐sensitive layer (top textile). c,d) Optical images of the top textile (c) before and (d) after processing from cross‐sectional view. e,f) The contact angle between water and top textile after the process of (e) hydrophobization and (f) nickel plating. g) SEM image of bottom textile before processing. h) SEM image of the Ni‐decorated bottom textile for interdigital electrode after processing. The inset of dashed rectangular indicates the enlarged view of local Ni‐decorated textile fibers. i,j) Optical images of the ATP (i) without and (j) with the application of pressure. k,l) Schematic illustration of the working principle of the ATP. m) Equivalent circuit of the ATP under the use of pressure.

The ATP is fabricated using an electroless plating process, and the fabrication flowchart is illustrated in **Figure** **2**a. The specific fabrication details can be found in the Experimental Section. The proposed pressure sensor consists of two layers of different textiles with designed structures (Figure 2b). The top layer, serving as the pressure‐sensitive layer, is a nickel‐decorated 3D mesh made of polyester material, while the bottom textile is composed of nylon and cotton. An interdigitated electrode of metallic nickel is deposited on the bottom textile. Optical images of cross‐sectional views of the top textile before and after nickel plating are displayed in Figure 2c,d, respectively. However, the top textile undergoes hydrophobization to form an effective pressure‐sensitive layer, and the specific preparation details can be found in the Experimental Section. The contact angles of the top textile with water, after hydrophobization and nickel plating, are respectively shown in Figure 2e,f. Furthermore, scanning electron microscope (SEM) images of the bottom textile before and after nickel plating are presented in Figure 2g,h, respectively. The inset in Figure 2h depicts a 2000× magnified SEM image after nickel plating. Notably, the optical images and SEM demonstrate that the textile weaving structure remains intact following the electroless nickel plating process, with uniform deposition of metallic nickel on the textile fibers.

The inner fibers of the top textile (i.e., the pressure‐sensitive layer) contact with each other and subsequently contact with the interdigital electrodes on the bottom textile, forming an effective pressure sensing unit. Figure 2i presents the optical image of ATP in its unstressed state, providing a visual representation of its initial configuration. When an applied pressure is exerted, the top textile of ATP undergoes compression (Figure 2j), resulting in a noticeable reduction in the thickness of mesh and gap between the internal fibers. This structural transformation is further illustrated through the cross‐sectional schematic diagrams, showcasing the working principle of the ATP, as portrayed in Figure 2k,L. Under external pressure, the loosely packed pressure‐sensitive layer of the ATP experiences compression, reducing its thickness and increasing the probability of internal fibers coming into contact with each other. This contact area expansion leads to a reduction in the resistance of the pressure‐sensitive layer. Simultaneously, the diffusion of the conductive path results in a rapid increase in current, affecting the overall resistivity or current change of the sensor. Figure 2m displays the equivalent circuit diagram. In the absence of pressure, the resistance of ATP (*R*) is equal to the resistance of the bottom textile:

(1) $$R=R_{e}+R_{t}+R_{e}$$

where *R*_e_ is the one‐sided resistance of the interdigitated electrodes, *R*_t_ is the resistance of the bottom textile, and the top textile is regarded as an insulator. When pressure is applied to the ATP:

(2) $$R=R_{e}+(R_{c}+R_{p}+R_{c})//R_{t}+R_{e}\approx R_{e}+R_{c}+R_{p}+R_{c}+R_{e}$$

where *R*_p_ is the resistance of the top textile under pressure, and *R*_c_ is the contact resistance between the single‐sided interdigitated electrode and the top textile. As external pressure increases, the top textile undergoes greater compression, leading to an increased likelihood of fiber‐to‐fiber contact within the middle portion of the textile. Consequently, ATP experiences an expansion of the contact area and a closer proximity between the fibers. This process ultimately leads to a reduction in resistance. Therefore, the electrical resistance (*R*_p_) of the top textile decreases with increasing pressure, resulting in an overall decrease in the electrical resistance of ATP.

**Figure 3:**
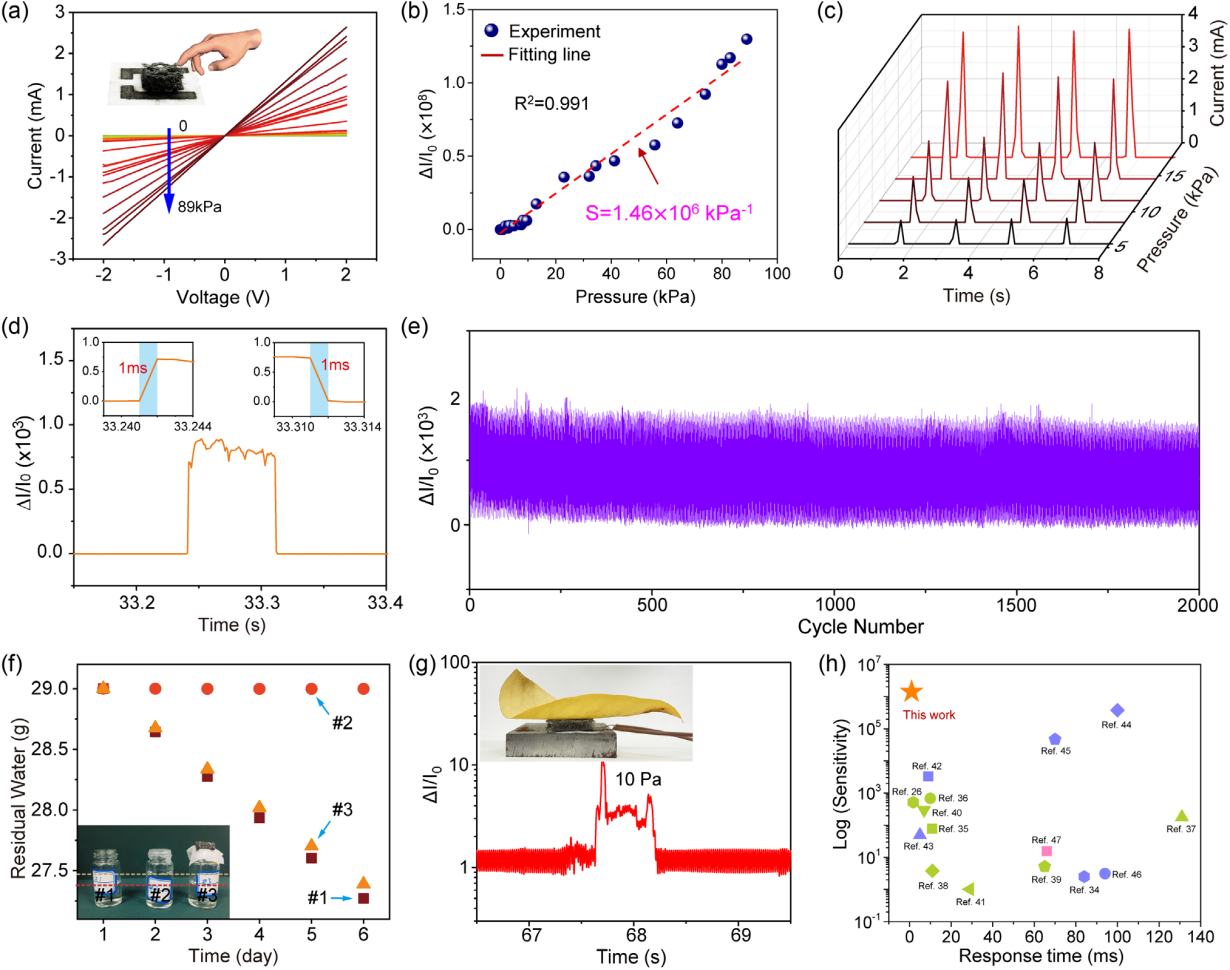
Device performance of ATPs. a) *I*–*V* curves of the ATPs under different pressure. b) Relative current change (Δ*I*/*I*_0_) as a function of pressure, indicating a record ultrahigh sensitivity of 1.46 × 10^6^ kPa^−1^. c) Temporal current changes of the ATPs under different pressures (voltage = 2 V). d) Fast temporal response to a small pressure. e) Endurance test of the ATPs over 2000 cycles under 1 kPa. f) Residual water weight as a function of time in the samples of #1 (no sealed), #2 (sealed with sealing film), and #3 (sealed with ATPs), showing excellent air permeability of the ATPs of 93.2%. g) Detection pressure limit of 10 Pa. h) Device performance comparison in different pressure sensors (the green shapes represent the resistive pressure sensors,^[^ ^50^, ^51^, ^52^, ^53^, ^54^, ^55^, ^56^ ^]^ the blue shapes represent capacitive pressure sensors,^[^ ^57^, ^58^, ^59^, ^60^, ^61^, ^62^ ^]^ and the pink shapes represent piezoelectric pressure sensors^[^ ^63^ ^]^).

Key parameters, such as stability, sensitivity, durability, and response time, are commonly used for evaluating the performance of pressure sensor. **Figure** **3**a shows the current–voltage (*I*–*V*) curves of the ATP under different pressures, ranging from 0 to 89 kPa. The *I*–*V* curves exhibit ultrahigh linearity, and the corresponding slope increases with the increase of applied pressure, suggesting that the pressure‐sensitive sensing response of ATP is stable under different external pressures. In addition, the sensitivity of a pressure sensor can be defined as *S* = (Δ*I*/*I*_0_)/Δ*P*, where Δ*I* is the relative change in current when a pressure *P* is applied, and *I*_0_ is the initial current before pressure is applied. Different external pressures were measured as a function of Δ*I*/*I*_0_ at a read voltage of 0.6 V, as shown in Figure 3b. Based on the linear fitting result, the ATP exhibits a record ultrahigh sensitivity of 1.46 × 10^6^ kPa^−1^ and high linearity (*R* ^2^ = 0.991) at the pressure range of 0–89 kPa. To further verify the reliability, four compression‐release cycles (at *V*_bias_ = 2 V) were performed on ATP under different external pressures of 5.5, 8.5, 11.5, 14.5, and 17.5 kPa, respectively, which are shown in Figure 3c. The ATP exhibits a stable and repeatable current response with various pressures, indicating good reversibility and stability of the device. And a fast response and relaxation time as small as 1 ms is observed in Figure 3d. Moreover, the ATP shows no significant drift or fluctuation during 2000 compression‐release cycles, demonstrating excellent mechanical durability for practical applications of pressure sensing (Figure 3e).

As a pressure sensor made of all‐textile material, air permeability is one of the important criteria to measure the wearing comfort. The gas permeability of the sensor was measured by evaporating water under natural conditions (Figure 3f). Filling three identical bottles (labeled #1, #2, and #3 respectively) with equal amounts of water, and sequentially leaving the bottle mouths uncovered, covered with parafilm, and capped with ATP, respectively. The free evaporation of water in bottle #1 was considered 100% air permeability, the sealing membrane on bottle #2 was considered 0 air permeability, and the air permeability of the ATP was calculated to be 93.2% after the bottle was left to sit for 6 days. The high air permeability of textile‐based ATP can contribute to enabling long‐term contact or attachment with human skin without causing mechanical damage or eczema to the human body. Moreover, upon placing a small piece of leaf on the ATP, the device has the ability to detect a very tiny pressure as low as 10 Pa, as shown in Figure 3g, which is very useful for the detection of some weak touch signals in wearable applications. When compared to the device performance of some previously reported pressure sensors, the ATP shows a higher sensitivity of 1.46 × 10^6^ kPa^−1^ and smaller response time of 1 ms, as shown in Figure 3h. Therefore, the textile‐based ATP, with excellent sensing capability and high air permeability, possesses distinctive advantages that make it particularly well suited for wearable applications.

**Figure 4:**
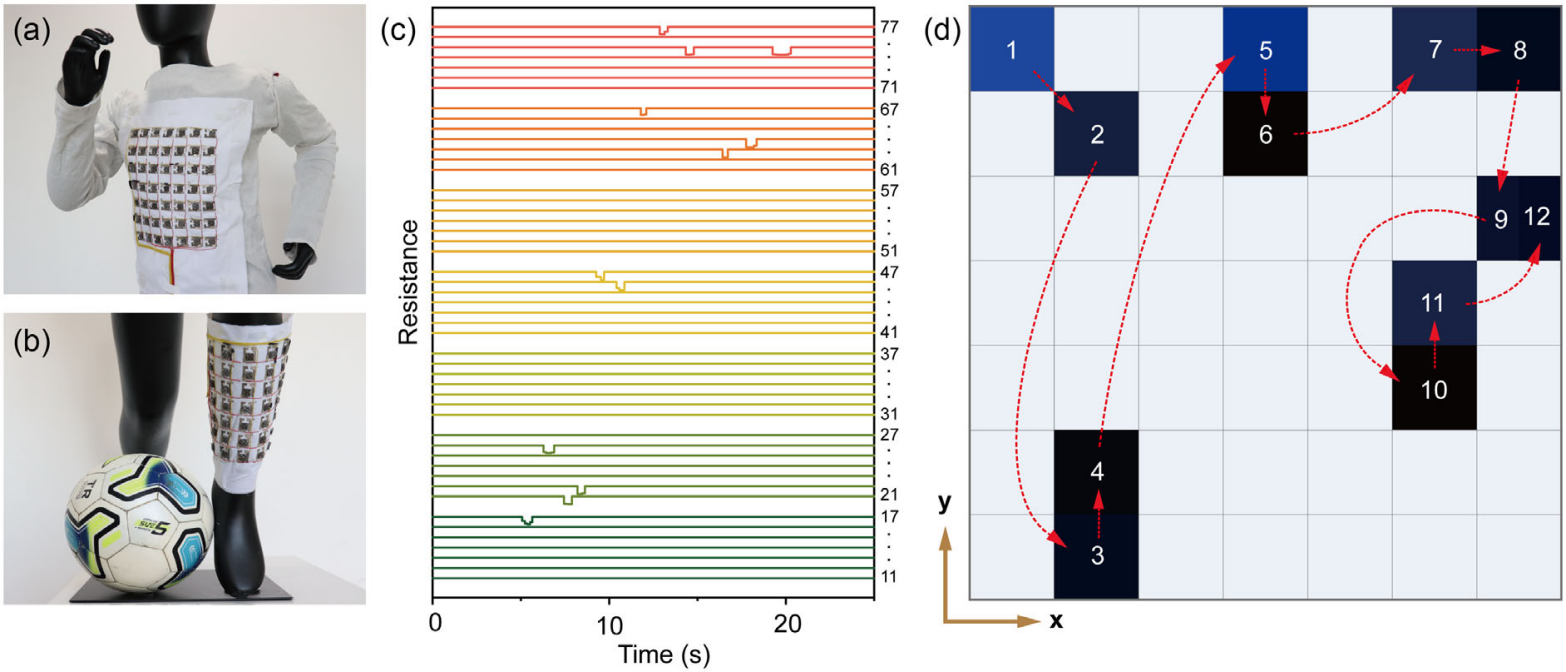
Body‐integrated dynamic pressure spatial mapping for interactive applications. a,b) Optical images showing the body‐integrated ATPs array in the applications of sports motion monitoring. c,d) Dynamic pressure spatial mapping for a trajectory monitoring. (c) Temporal resistance changes in a 7 × 7 channels of the ATPs array. (d) Contour profile of the trajectory of finger touch.

To demonstrate the practical application of ATP in real‐time tactile sensing for human–computer interaction, a 7 × 7 sensor array of ATP is fabricated. This array enables static and dynamic spatial pressure sensing and imaging. A customized data acquisition module was utilized to transmit real‐time tactile feedback information (e.g., pixel resistance values) to a computer for analysis via Bluetooth low energy technology. The major advantages of textile‐based sensors, such as excellent flexibility, comfort, and breathability, allow for their arbitrary customization, much like clothing. These sensors can be harmlessly attached to various positions on the human body. As shown in **Figure** **4**a,b, the ATP‐based array perfectly fits on the front chest and calf of the mannequin. Additionally, the ATP‐based array demonstrates the ability to monitor pressure trajectories, which is recorded in Movie S1, Supporting Information. Figure 4c presents the sequence of resistance changes in each pixel of the 7 × 7 array, and Figure 4d displays the corresponding spatial mapping pattern of a finger touch trajectory. The ATP‐based array not only enables real‐time touch input recognition, but also allows for changes in color depth within the spatial mapping pattern based on external pressure. It enables simultaneous and accurate monitoring of the magnitude, position, and imaging of the applied pressure. Consequently, these results emphasize the significant advantage of the ATP in wearable smart sensing and monitoring devices. The proposed all‐textile pressure sensor holds immense application potential in human–computer interaction applications, such as motion monitoring, physiological health monitoring, and VR, highlighting the promising future of ATP in enhancing user experiences and advancing wearable technology.

**Figure 5:**
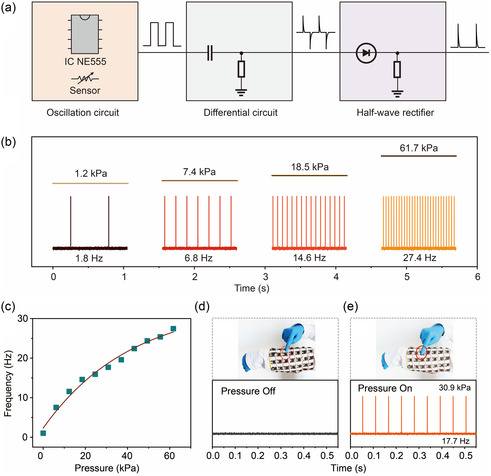
Neuron‐like spiking behavior of the ATPs. a) Schematic diagram of the circuit design, including a NE555 oscillation circuit, a differential circuit, and a half‐wave rectifier, to process analog signals of a sensor into spiking frequency signals. b) The converted spiking frequency under different pressures of 1.2, 7.4, 18.5, and 61.7 kPa. c) Frequency response as a function of applied pressure. d,e) The generated frequency signals response to two conditions of finger touch pressure on one pixel of an ATP array: (d) no pressure and (e) under a pressure corresponding to 30.9 kPa. Optical images illustrate the two conditions of finger touch pressure on one pixel of the ATP array.

While ATP exhibits excellent performance, its true potential in achieving real human–computer interaction relies on establishing a direct link between the sensors and human neurons. Thus, a crucial aspect is converting the analog signal generated by the sensor into a physiological signal (in the frequency domain) recognizable by human neurons. To further explore the practical applicability of the sensor, an oscillation circuit was constructed by using ATP and NE555 oscillator, which enables the conversion of pressure stimulations into digital frequency signals. The working principle of the circuit system is illustrated in **Figure** **5**a. The ATP serves as a variable resistor and is used to form an oscillation circuit with the NE555 oscillator to generate square wave signals. The external pressure stimulation can modify the resistance of ATP, thereby adjusting the frequency of the output signals. Subsequently, the square wave signal undergoes conversion into a frequency‐adjustable pulse waveform resembling human nerve signal through an integration and filtering circuit. The pulse waveforms, which dynamically change with the applied pressure, are considered as the output of the system. By implementing this approach, the ATP can effectively translate physical pressure stimuli into frequency‐modulated signals, bridging the gap between the sensor output and human neural perception. This advancement marks a significant step toward realizing seamless human–computer interaction using ATP‐based sensors.

Figure 5b demonstrates the output signals obtained under loadings of 1.2, 7.4, 18.5, and 61.7 kPa, showcasing an increase in the number of output pulses corresponding to the applied pressure. This behavior closely resembles human tactile perception. To further explore the relationship between external pressure stimuli and frequency response, Figure 5c presents the results. It reveals that the frequency of output pulses increases with pressure within the range of 0–61.7 kPa. By fitting the experimental data, an exponential function $f=33.77-31.47e^{-P/41.76}$ can be obtained, where *f* represents the frequency response (Hz) and *P* is the pressure stimulus (kPa). The fitting result is consistent with the stimulus‐response function,^[^ ^46^ ^]^ providing a comprehensive understanding of the system's behavior. In a practical demonstration, the ATP‐based array is worn on the human arm and connected to the signal conversion circuit, resulting in the construction of an intelligent interactive cuff that seamlessly integrates stimulation sensing and neuron encoding functions. Figure 5d,e shows the corresponding output pulse waveforms before and after finger pressing, respectively, while the upper panel displays photos capturing these operations. When a load is applied to the sensor, a 17.7 Hz digital pulse waveform, closely resembling that of a human, is obtained. If ATP can 57technology, it would eliminate the need for bulky equipment. Instead, individuals would only require the wearing of these extraordinary “clothes” to immerse themselves in surreal VR games. Additionally, the proposed ATP holds promise for replacing damaged skin and finding applications in prosthetics, expanding its potential impact in the field of healthcare.

**Figure 6:**
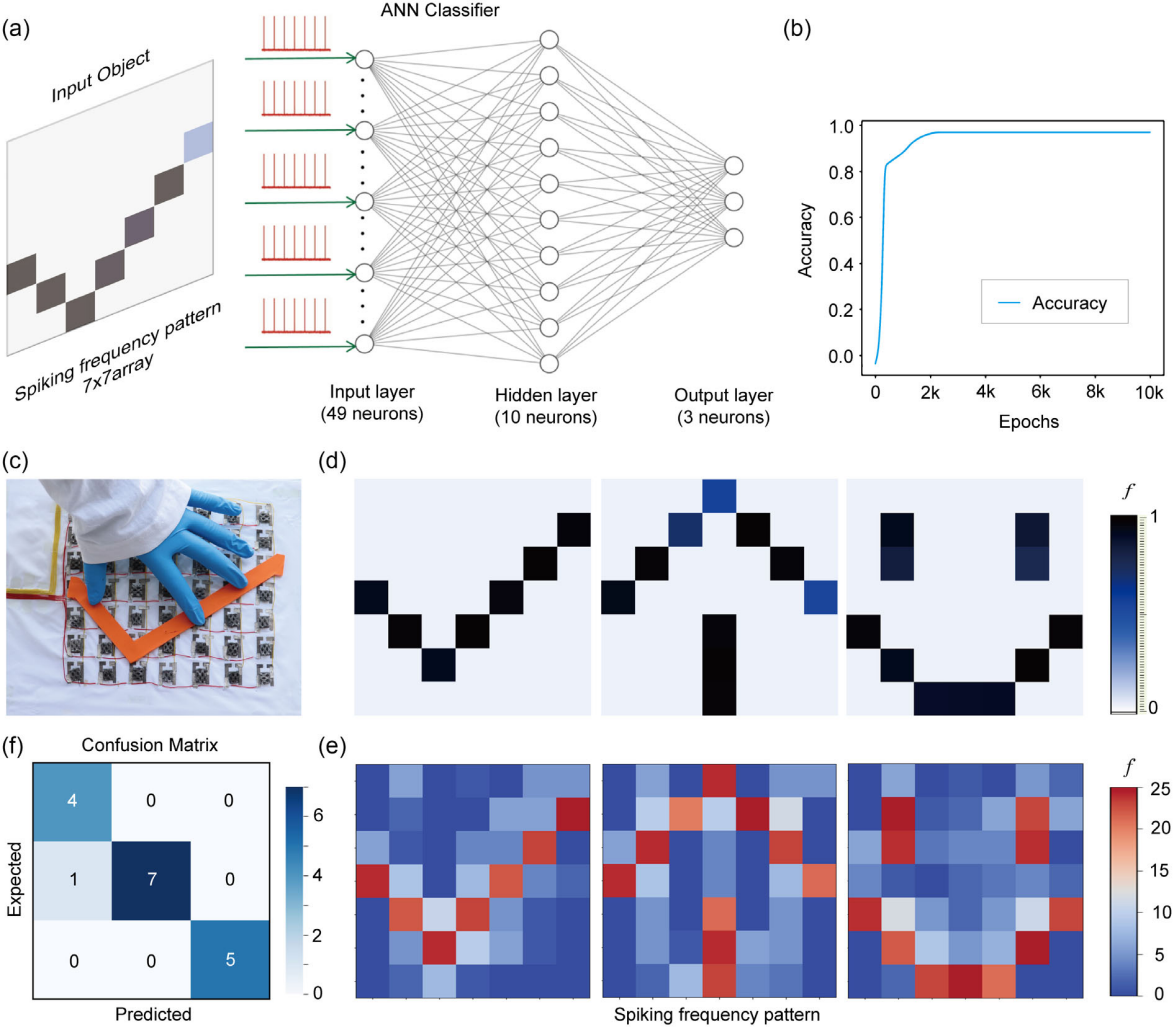
ATP‐based artificial sensory system for pressure recognition. a) Schematic of a constructed ANN for simulation. b) Evolution of recognition accuracy with training epochs. c) Optical image illustrating a hook‐shaped stamp statically pressed on the ATP array. d) Corresponding pressure spatial mappings, represented in a normalized frequency form, are illustrated for different stamp patterns: hook (left), row (middle), and smiling (right). e) Simulation of three shaped patterns under different pressures, which are corresponding to spiking frequency signals. f) Confusion matrix of simulation classification output versus the expected outputs.

To achieve pressure‐frequency capability of tactile perception, a three‐layer artificial neural network (ANN) model is constructed, consisting of an input layer, hidden layer, and output layer.^[^ ^47^, ^48^, ^49^ ^]^ The ANN model for pressure‐frequency recognition, depicted in **Figure** **6**a, comprises 49 input neurons, 10 hidden neurons, and 3 output neurons. Initially, 40 data samples are generated, with half allocated for training and the remaining for testing purposes. Simulation results demonstrate that the system achieves over 90% accuracy in pressure recognition, as shown in Figure 6b. Figure 6c presents the optical image of a “hook” pattern loaded on the ATP array, while the corresponding spatial pressure map of “hook”, “arrow”, and “smiling” patterns is observed in Figure 6d. Real‐time static and dynamic pressure sensing process using the ATP‐based array is depicted in Movie S2, Supporting Information. Each pixel in the ATP‐based array exhibits a distinct change in resistance, corresponding to frequency, thereby enabling real‐time identification of object shapes in response to the applied pressure. Based on these experimental results, an object classification task is performed to recognize three patterns with different shapes (“hook,” “arrow,” and “smiling”) in the 7 × 7 array, as shown in Figure 6e. To achieve the pressure pattern recognition, the collected datasets include 49 pressure signals carrying position information from the 7 × 7 sensor array. The collected data represent different pattern pixels corresponding to “hook,” “arrow,” and “smiling.” During simulation, patterns in various states are placed on the array for testing, generating frequency signals from the 49 sensors. Notably, when multiple parallel pressure sensors acquire frequency data, it becomes challenging to interpret their specific meaning. Thus, the specific data are presented in the form of a pressure graph (Figure 6e), and the system can autonomously adjust the threshold to facilitate confirmation of object pressure information. The confusion matrix (Figure 6f) illustrates the simulation's classification capability output compared to the expected output (the color bar reflects the count of the output neuron), showcasing the excellent classification capability of the ATP array. Remarkably, the ATP‐based artificial sensory system can discriminate between objects with different pressure levels using frequency signals, akin to the functioning of the biological somatosensory system. This demonstrates the immense potential of ATP arrays in constructing intelligent sensory systems.

## 3 Conclusion

In summary, a resistive pressure sensor with a double‐layer structure was fabricated by employing a straightforward electroless plating process to deposit metallic nickel into the textile. The pressure sensitivity of the sensor relies on the resistance decrease of the upper textile as the pressure increases. The ATP exhibits remarkable characteristics, including ultrahigh sensitivity (1.46 × 10^6^ kPa^−1^), ultrafast response time and relaxation time (1 ms), excellent stability and durability (up to 2000 compression–release cycles), and a low detection limit (10 Pa). Furthermore, the ATP shows a remarkable gas permeability of 93.2%. Thanks to its all‐textile construction, the ATP can be harmlessly attached to various parts of the mannequin, such as the chest and lower legs, in any desired shape without causing harm. Leveraging data acquisition hardware circuitry and Bluetooth communication technology, the 7 × 7 pressure sensor array based on ATPs successfully achieves spatial pressure mapping and pressure trajectory monitoring. Additionally, a signal conversion circuit is constructed using the ATP as a variable resistor, enabling the encoding of force stimulation signals applied to ATP into digital frequency pulses akin to physiological signals. The integration of the ATP‐based array with the signal conversion circuit effectively demonstrates an intelligent interactive sleeve capable of simulating skin perception and interaction functions. The proposed all‐textile pressure sensor boasts excellent performance, making it highly suitable for wearable electronics. By mimicking the capabilities of human skin, the ATP‐based sensor array contributes toward the development of advanced wearable devices that enhance our interaction with the world around us. It holds tremendous potential for creating innovative applications in fields such as motion monitoring, electronic skin, and VR.

## 4 Experimental Section

4.1

4.1.1

##### Materials of ATPs

ATPs were made up of two textiles of different materials and structures. Sandwich‐type 3D mesh textile, made of polyester material with a thickness of about 4 mm and a mesh size of 2 mm × 3 mm (length × width), is employed to serve as the pressure‐sensitive material of top textile. The textile woven together with nylon and cotton fiber is used as the bottom textile. The density is 310 T, the yarn is 40 D, and the weight is about 53 g per square meter. It has a somewhat hydrophobic property.

##### Fabrication of ATPs

The two commercial textiles area first cut, and then the two textiles are ultrasonic cleaned in acetone solution, alcohol solution, and deionized water for 5 min at a time. Finally, the textiles are dried with a hair dryer.

Due to the top textile poor hydrophobicity, it should be treated with hydrophobicity before electrical modification. The top textile is soaked in 1% silane coupling agent (KH560) ethanol solution for 3 min, and then dried on a heating table at 100 °C, repeated 5 times, and the hydrophobic process is completed (Figure 2e shows the contact angle between the top textile and water after hydrophobic is 47°).

Use Kapton tape to tightly cover both sides of the hydrophobic top textile. This method allows the thickness of the metal nickel deposited on the textile to vary from the middle to the edge of the textile during deposition. Nickel is then deposited on the textile by electroless plating deposition; the process is as follows.

The top textile with a mask is soaked in acidified 10 g L^−1^ SnCl_2_ solution and 0.5 g L^−1^ PdCl_2_ solution for 90 s, respectively. Each solution is soaked 3 times. After each soak, remove and rinse with deionized water, and then dry with a hair dryer. The preserved mask of the soaked textile is soaked for 30 min in a bath prepared in advance (a mixture of 17.52 g L^−1^ NiSO_4_⋅6H_2_O, 25 g L^−1^ NaH_2_PO_2_⋅H_2_O, 15 g L^−1^ C_6_H_5_Na_3_O_7_⋅2H_2_O, 30 g L^−1^ H_3_BO_3_). Rinse with deionized water and dry with a hair dryer. The Kapton tape is removed and the top textile is finished (Figure 2f shows the contact angle between the top textile and water after nickel plating is 52°).

The bottom textile is tightly covered with Kapton tape over both sides. Then, interdigitated electrodes are cut out on the tape using a CO_2_ laser cutter, and the specific dimensions of the electrodes are shown in Figure 2a. Under the patterned tape mask, nickel plating without chemical deposition is carried out on the textile, which is the same as the above experimental process. The only difference is that the treatment of the bottom textile is soaked in the plating solution for 6 h. After soaking for 6 h, remove it, rinse it with deionized water, dry it with a hair dryer, and remove the mask tape.

The top textile is further cut to the size of the interdigital electrode of the bottom textile, and the two are fixed together with a needle and thread. The top textile has the mesh side up and the other side is next to the bottom textile. The ATPs are prepared, and the wires are drawn on both sides of the interdigital electrode for the subsequent performance test. The whole preparation process of the ATP is shown in Figure 2a.

##### Preparation of ATP‐Based Array

With the same textile as the bottom textile as the array base, 49 ATPs are fixed on the textile to form a 7 × 7 array, and the wires are extracted and sorted out.

##### Structural Characterization and the Performance of ATPs

The structure of the underlying textile was studied by SEM (Hitachi SU8020). The *I*–*V* curve and sensitivity were tested using a semiconductor device analyzer equipped (Keysight B1500A) with EasyEXPERT group+ software. Mechanical cycle stability was tested using a linear motor (Linmot E1100) and an electrochemical workstation (CHI660E). Frequency test was conducted using an oscilloscope (TELEDYNE LECROY, HD06104).

##### Air Permeability Test

First, prepare three glass bottles of the same size, wash them with deionized water, and put them into a constant temperature drying oven at 60 °C to dry the water. After labeling them (#1, #2, and #3), they are weighed before water injection. After adding the same amount of water, they are weighed again, and the weight of the initial water in the bottle is calculated. The #1 bottle whose mouth is not covered acts as a control sample. The #2 bottle was tightly sealed with the sealing film, and the #3 bottle was covered with ATPs. The sensor is tightly attached to the bottle under the condition that the double‐sided glue does not block the bottle. Quickly complete the above sealing operation and weigh the three glass bottles again. The weight of water lost during the sealing process was ignored. The glass bottles were kept indoors under natural conditions, and for the next 5 days, the glass bottles were weighed at the same time to calculate the remaining water volume.

##### Stimulation of the ATP‐Based Artificial Sensory System

The ATP‐based artificial sensory system for pressure pattern recognition using the artificial tactile sensory neurons is implemented in simulation by the Pytorch based on experimental data. During the training process, the optimization of the network followed the standard back propagation of errors with a rectified linear unit activation function in the hidden layer and a sigmoid activation function in the output layer.^[^ ^47^, ^48^, ^49^ ^]^ During the inference process, the output pressure depends on the input frequency signal.

## Conflict of Interest

The authors declare no conflict of interest.

## Supporting information

Supplementary Material

## Data Availability

The data that support the findings of this study are available from the corresponding author upon reasonable request.
